# TRAF2 protects against cerebral ischemia-induced brain injury by suppressing necroptosis

**DOI:** 10.1038/s41419-019-1558-5

**Published:** 2019-04-15

**Authors:** Jie Li, Jingyu Zhang, Yusuo Zhang, Zichuang Wang, Yanmei Song, Shanwen Wei, Meijun He, Shoujiang You, Jia Jia, Jian Cheng

**Affiliations:** 1 Department of Neurology and Suzhou Clinical Research Center of Neurological Disease, The Second Affiliated Hospital of Soochow University, Soochow University, Jiangsu Province, Suzhou, China; 2 Jiangsu Key Laboratory of Neuropsychiatric Diseases and Institute of Neuroscience, Soochow University, Jiangsu Province, Suzhou, China; 3 Jiangsu Key Laboratory of Neuropsychiatric Diseases and College of Pharmaceutical Sciences, Soochow University, Jiangsu Province, Suzhou, China

## Abstract

Necroptosis contributes to ischemia-induced brain injury. Tumor necrosis factor (TNF) receptor associated factor 2 (TRAF2) has been reported to suppress necroptotic cell death under several pathological conditions. In this study, we investigated the role of TRAF2 in experimental stroke using a mouse middle cerebral artery occlusion (MCAO) model and in vitro cellular models. TRAF2 expression in the ischemic brain was assessed with western blot and real-time RT-PCR. Gene knockdown of TRAF2 by lentivirus was utilized to investigate the role of TRAF2 in stroke outcomes. The expression of TRAF2 was significantly induced in the ischemic brain at 24 h after reperfusion, and neurons and microglia were two of the cellular sources of TRAF2 induction. Striatal knockdown of TRAF2 increased infarction size, cell death, microglial activation and the expression of pro-inflammatory markers at 24 h after reperfusion. TRAF2 expression and necroptosis were induced in mouse primary microglia treated with conditioned medium collected from neurons subject to oxygen and glucose deprivation (OGD) and in TNFα-treated mouse hippocampal neuronal HT-22 cells in the presence of the pan-caspase inhibitor Z-VAD. In addition, TRAF2 knockdown exacerbated microglial cell death and neuronal cell death under these conditions. Moreover, pre-treatment with a specific necroptosis inhibitor necrostatin-1 (nec-1) suppressed the cell death exacerbated by TRAF2 knockdown in the brain following MCAO, indicating that TRAF2 impacted ischemic brain damage through necroptosis mechanism. Taken together, our results demonstrate that TRAF2 is a novel regulator of cerebral ischemic injury.

## Introduction

Stroke is a leading cause of mortality and disability worldwide^1^, and ischemic stroke accounts for >80% of total stroke. As ischemia damages the brain tissue by deprivation of oxygen and metabolic substrates, danger signals are released from cells under ischemic stress. The danger associated molecular pattern molecules (DAMPs) activate pattern recognition receptors on microglia and induce an inflammatory response by expressing pro-inflammatory mediators, such as tumor necrosis factor α (TNFα), resulting in inflammation-induced necrotic neuronal cell death^2^. Neuronal necrosis and loss of interaction between neurons and microglia further promote the inflammatory signaling^2^, thus exacerbate the ischemic brain injury^3^. Necroptosis is a form of programmed necrosis, and mounting evidence has shown that necroptosis is of great pathophysiological relevance in ischemic brain injury^4–10^. Inhibition of necroptosis significantly reduces infarct volumes^4,5,7^, attenuates inflammatory response^8^, improves locomotive ability^9^ and cognitive function^8,9^ following cerebral ischemia.

The initiation of necroptosis requires the kinase activity of receptor-interacting protein 1 (RIP1). The execution of necroptosis is comprised of the activation of the receptor-interacting protein 3 (RIP3) by RIP1, and subsequent phosphorylation and oligomerization of mixed lineage kinase domain-like (MLKL), allowing the association of MLKL with phospholipid membranes, and ultimately leading to membrane disruption and necroptotic cell death^11^. Recently, tumor necrosis factor (TNF) receptor associated factor 2 (TRAF2) has been reported to suppress necroptosis through different mechanisms. TRAF2 could suppress death receptor-triggered necroptosis by recruiting cIAP1/2^12^, and suppress TNFα plus cycloheximide plus Z-VAD-FMK (Z-VAD)-induced necroptosis by directly binding to MLKL and restricting the association of MLKL with RIP3^13^. The TRAF protein family consists of seven members (TRAF1-7), with TRAF1 and TRAF2 as the adaptor proteins of TNF receptor 2. Interestingly, TRAF1 expression is markedly induced, and TRAF1 promotes neuronal death and exacerbates damage following cerebral ischemia^14^. However, whether TRAF2 participates in the brain pathology following cerebral ischemia remains unclear.

## Materials and methods

### Materials

DMEM, DMEM/F12 were purchased from HyClone (Beijing, China). Fetal bovine serum, neurobasal medium and B27 were purchased from Gibco (Grand Island, NY, USA). M-CSF was purchased from Peprotech (Rocky Hill, NJ, USA). Lentiviruses expressing non-targeted control (NC) short hairpin RNA (shRNA) or TRAF2 shRNA were provided by GeneChem (Shanghai, China). Z-VAD was purchased from BD Biosciences (San Jose, CA). TNFα was purchased from Sigma-Aldrich (St. Louis, MO, USA). Propidium iodide (PI) was purchased from Sangon Biotech (Shanghai, China). RIPA lysis buffer, NP-40 lysis buffer and DAPI were purchased from Beyotime (Shanghai, China). Protease inhibitors cocktail was purchased from Roche (Indianapolis, IN, USA). BCA protein assay kit was purchased from Pierce (Rockford, IL, USA). The primary antibodies were: anti-TRAF2 (Santa Cruz Biotechnology, Santa Cruz, CA, USA), anti-NeuN (Millipore, Billerica, MA, USA), anti-Iba1 (Wako, Richmond, VA, USA), anti-MLKL (Millipore, Billerica, MA, USA), anti-RIP1 (BD Biosciences, San Jose, CA, USA), anti-RIP3 (ProSci Incorporated, Poway, CA, USA) and anti-β-actin (Multi Sciences Biotech, Hangzhou, China). The secondary antibodies were: HRP-conjugated anti-mouse IgG (Multi Sciences Biotech, Hangzhou, China), HRP-conjugated anti-rabbit IgG (Sigma-Aldrich, St. Louis, MO, USA), HRP-conjugated anti-rat IgG (Multi Sciences Biotech, Hangzhou, China), Alexa Fluor 555-conjugated goat-anti-rabbit IgG (Invitrogen, Carlsbad, CA, USA), Alexa Fluor 488-conjugated goat anti-mouse IgG (Invitrogen, Carlsbad, CA, USA). Necrostatin-1 (nec-1), normal rat IgG and protein A/G PLUS-agarose were purchased from Santa Cruz Biotechnology (Santa Cruz, CA, USA). Calcein AM was purchased from Life Technologies (Grand Island, NY, USA). RNA extraction kit was purchased from TIANGEN (Beijing, China). Revert Aid M-MuLV reverse-transcriptase was purchased from Fermentas (Burlington, Canada). SYBR green master mix was purchased from ABI (Foster City, CA, USA).

### Animals

Adult male ICR mice weighing 25–30 g were purchased from SLAC Laboratory Animal Co. (Shanghai, China). All the animal procedures were approved by the Animal Care and Use Committee of Soochow University.

### Transient middle cerebral artery occlusion (MCAO), lentivirus infection and drug administration

Mice received 1 h of MCAO, as previously described^3,15–18^ with some modifications. In brief, mice were anesthetized by isoflurane mixed with oxygen and air. Right common carotid artery was separated and ligated with medical suture, and right internal carotid artery and external carotid artery were separated. At the external carotid artery site 1 cm to the internal and external cervical vascular branch, ligation was carried out. A small cut was made at the artery site close to the ligation and proximal to the heart, and a nylon monofilament with its tip coated with silicon and heat-blunted was inserted into the right internal carotid artery through the cut. Laser Doppler flowmetry was used to monitor the right brain artery blood flow. The filament was secured when its tip reached the origin of the middle cerebral artery, as indicated by an apparent drop (~30% of baseline) in cortical blood flow. After 1 h of occlusion, the filament was extracted to allow for reperfusion, and blood flow was recovered to >70% of baseline. Sham-operated mice underwent the same surgery except for filament insertion. Mice were maintained on top of a warming pad during the above procedures. In total, MCAO was successfully induced in 86 mice that were used in further experiments. Another 15 mice died within 24 h after reperfusion and were excluded from the experiments.

In some experiments, two weeks before MCAO, two injections of NC shRNA lentivirus or TRAF2 shRNA lentivirus (1 μl of 5 × 10^8^ TU/ml lentivirus per injection)^19^ were injected into the ipsilateral striatum of the mice.

In some experiments, vehicle (DMSO) or nec-1 (3 μl, 4 mM)^4^ was injected into the ipsilateral cerebral ventricle of the mice, and MCAO was immediately carried out.

### Immunofluorescence microscopy

At 24 h after reperfusion, mice were euthanized, perfused with saline and fixed with 4% paraformaldehyde (PFA). The brains were collected, fixed with 4% PFA and dehydrated with 30% sucrose. Then the brain blocks surrounded the MCA territory (+1.18 to −0.10 mm relative to bregma) were cut into 10 μm-thick coronal sections with a cryostat, and four brain slices separated by 150 μm were used for immunofluorescence staining. Slices were blocked with PBS containing 3% BSA, 10% normal goat serum and 0.3% Triton X-100 and incubated with primary antibodies overnight. On the next day, slices were incubated with corresponding secondary antibodies conjugated to Alexa Fluor. Images from each section containing three pre-assigned cortical fields and three pre-assigned striatal fields inside the lesion core in the ischemic brain were taken by confocal microscopy (Zeiss LSM700, Germany). The amounts of single-positive (Iba1^+^, NeuN^+^, TRAF2^+^) and double-positive (TRAF2^+^Iba1^+^, TRAF2^+^NeuN^+^) cells were manually counted, and the percentages of double-stained cells to single-stained cells were calculated.

### TTC staining and infarct volume assessment

The procedures were as described previously^3^. Briefly, at 24 h after reperfusion, mice were sacrificed and the brains were cut into four 2 mm-thick slices. Sections were stained with 1% 2, 3, 5-tripheyltetrazolium chloride (TTC) (Sigma, St. Louis, MO, USA) for 10 min at 37 °C, and fixed in 10 % PFA overnight. The images were taken by a digital camera, and the infarct volumes were blindly analyzed with SigmaScan Pro software (Jandel, San Rafael, CA, USA). Infarct sizes were expressed as percentages of the contralateral structures^19^.

### PI staining

At 21 h after reperfusion, mice were intraperitoneally injected with vehicle (saline) or PI (20 mg/kg). After 3 h, mice were killed, perfused with saline and fixed with 4% PFA. The brains were collected, fixed with 4% PFA and dehydrated with 30% sucrose, and the brain blocks surrounded the MCA territory (+1.18 to −0.10 mm relative to bregma) were cut into 20 μm-thick coronal sections with a cryostat. Images were captured with fluorescence microscopy (Zeiss Axio Scope A1, Germany). PI^+^ cells in three random 200 × striatal fields from each section and four sections adjacent to the lesion core and separated by 150 μm from each mouse brain were analyzed. The average amount of PI^+^ cells in a striatal field for a given sample was calculated by summing the amounts of PI^+^ cells from all the fields and divided by twelve.

### Cell culture and oxygen-glucose deprivation (OGD) treatment

Primary microglia were obtained from newborn wildtype C57BL/6J mice at post-natal day 1-2, as previously described^3^. Primary cortical neurons were obtained from 16/17-day old embryos of wildtype C57BL/6J mice and subjected to OGD at DIV 10, similarly as previously described^20–24^. At 24 h after re-oxygenation, the conditioned media (OGD neuron CM) were collected, aliquoted and stored in the −80 °C freezer for later use. Media collected from neurons without OGD treatment served as the control.

Hippocampal neuronal HT-22 cells were cultured in DMEM medium supplemented with 10% fetal bovine serum and 1% penicillin/streptomycin and maintained at 37 °C in humidified air containing 5% CO_2_.

### Lentivirus infection, drug treatment and cell staining

Primary microglia or HT-22 cells were infected with NC shRNA or TRAF2 shRNA lentivirus (5 × 10^5^ TU/well for 24-well plate). At 4-5 days after infection, microglia were treated with OGD neuron CM in the absence or presence of 25 μM Z-VAD with/without 20 μM nec-1 for 12 h, and HT-22 cells were treated with 1 μg/ml TNFα in the absence or presence of 25 μM Z-VAD with/without 20 μM nec-1 for 12 h. After treatment, cells were stained with 5 μM PI and 2 μM Calcein AM for 15 min and observed by confocal microscopy (Zeiss LSM700, Germany). The amounts of PI^+^ cells were manually quantified, and the ratio of PI^+^ cells compared to control was regarded as cell death.

### Western blot

The procedures were as described previously^3^. In brief, tissue or cell samples were lysed in RIPA lysis buffer and centrifuged to remove cell debris. Proteins (40 μg/sample) were separated on SDS-PAGE gels and transferred to PVDF membranes. After blocking, the membranes were sequentially incubated with primary antibodies (anti-TRAF2, anti-RIP1, anti-RIP3, anti-MLKL, anti-β-actin) and HRP-conjugated secondary antibodies, and the protein bands were visualized with Immobilon Western Chemiluminescent HRP substrate (Merck Millipore, Billerica, MA, USA) and captured with a Chemiluminescence Imaging System (ChemiDoc XRS+, Bio-Rad, CA, USA) using Image Lab software (Bio-Rad, CA, USA). β-actin served as the loading control. The optical densities of protein bands were semi-quantified by ImageJ software (NIH, Bethesda, MD, USA), and results were expressed as the ratio of target protein to β-actin.

### Co-immunoprecipitation

Striatum samples were lysed in NP-40 lysis buffer on ice for 30 min, and then centrifuged to remove the debris. The supernatant was transferred to a new tube, and protein concentration was determined using a BCA kit. 1 μg of anti-MLKL antibody or normal rat IgG was added into cell lysate containing 1 mg of total protein, and the tube was incubated with gentle rotating overnight at 4 °C. On the following day, 20 μl of Protein A/G agarose beads were added into the tube, and incubated with gentle rotating for 3 h at 4 °C. The beads were washed with lysis buffer for four times, and the supernatant was discarded. 20 μl of 2 × protein loading buffer was added to the beads, mixed and boiled for 5 min. The supernatant was collected for western blot analysis.

### Real-time RT-PCR

Procedures were as previously described^3^. Total RNA was extracted from tissue samples using an RNAprep pure Tissue kit (TIANGEN Biotech, Beijing, China) following the manufacturer’s instructions. The quantity and quality of the RNA was assessed with Nanodrop (Thermo Fisher Scientific, Wilmington, DE, USA). cDNA was reverse transcribed using a cDNA synthesis kit (Applied Biosystem, Foster City, CA, USA). Real-time PCR was performed on 7500 Sequence Detection System using SYBR green. The sequences of primers are as follows (5′ to 3′): 18 S RNA: forward: GTAACCCGTTGAACCCCATT, reverse: CCATCCAATCGGTAGTAGCG. Mouse TRAF2: forward: CCTACTGCTGAGCTCATTCT, reverse: CAATCTTGTCCTGGTCTAGC. Mouse iNOS: forward: CAGGAGGAGAGAGATCCGATTTA, reverse: GCATTAGCATGGAAGCAAAGA. Mouse TNFα: forward: CATCTTCTCAAAATTCGAGTGACAA, reverse: TGGGAGTAGACAAGGTACAACCC. Mouse CD32: forward: AATCCTGCCGTTCCTACTGATC, reverse: GTGTCACCGTGTCTTCCTTGAG. Results were normalized to 18 S RNA.

### Statistical analysis

The statistical analysis was performed using GraphPad Prism software. Data were analyzed by one-way ANOVA with Tukey’s post hoc test or by two-way ANOVA with Bonferroni post hoc test. All data were expressed as mean ± SEM, and *p* < 0.05 was considered statistically significant.

## Results

### TRAF2 was induced following cerebral ischemia

**Fig. 1:**
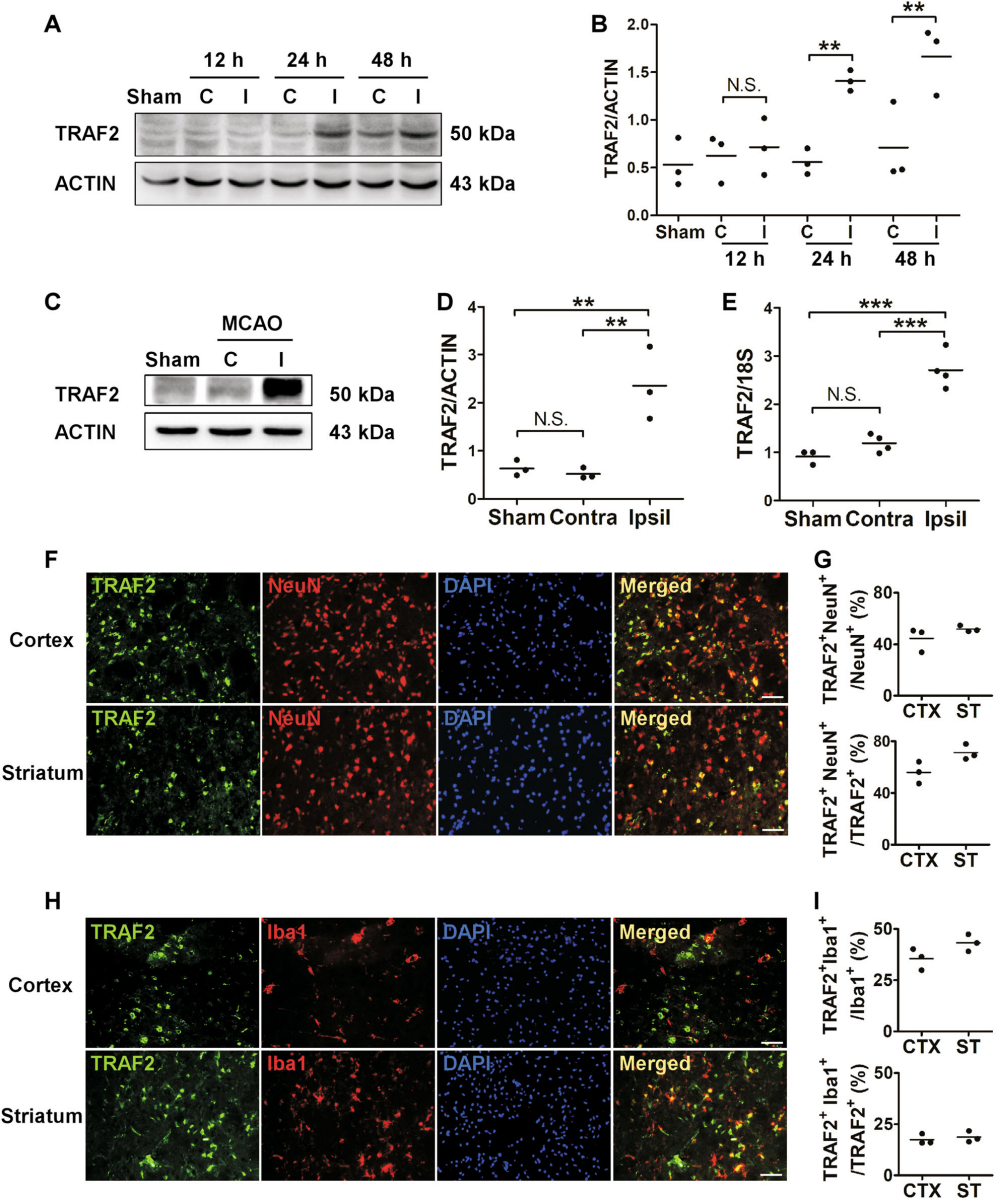
TRAF2 expression was induced in neuron and microglia at 24 h following reperfusion in a mouse MCAO model. **a** TRAF2 protein levels in the contralateral and ipsilateral cortex at 12, 24 and 48 h after reperfusion. β-actin served as the internal reference. Sham: sham-operated mice. C: contralateral side. I: ipsilateral side. **b** Quantification data of western blot analysis of TRAF2 protein (*n* = 3). N.S.: not significant; **p < 0.01. **c**, **d** Representative images and quantification of western blot analysis of the striatal expression of TRAF2 at 24 h after reperfusion (*n* = 3). N.S.: not significant; **p < 0.01. **e** The striatal mRNA levels of TRAF2 in sham-operated or MCAO mice at 24 h after reperfusion (*n* = 4). N.S.: not significant; ***p < 0.001. **f** Co-localization of TRAF2 with the neuronal marker NeuN in the ischemic cortex and striatum at 24 h after reperfusion. Scale bar: 50 μm. **g** Quantification data showing the percentages of TRAF2^+^NeuN^+^ cells to NeuN^+^ or TRAF2^+^ cells in the cortex (*n* = 3) and striatum (*n* = 3). **h** Co-localization of TRAF2 with the microglial marker Iba1 in the ischemic cortex and striatum at 24 h after reperfusion. Scale bar: 50 μm. **i** Quantification data showing the percentages of TRAF2^+^Iba1^+^ cells to Iba1^+^ or TRAF2^+^ cells in the cortex (*n* = 3) and striatum (*n* = 3)

We used a well-established MCAO and reperfusion injury model to examine the expression pattern of TRAF2 following cerebral ischemia. Compared to its expression in the contralateral cortex of mice received MCAO or that in the cortex of sham-operated mice, TRAF2 was significantly increased in the ipsilateral cortex, starting at 24 h after reperfusion (Fig. 1a, b). Consistently, TRAF2 expression was increased at the protein (Fig. 1c, d) and mRNA (Fig. 1e) levels in the ipsilateral striatum at 24 h after reperfusion. Immunofluorescence results further revealed that TRAF2 expression was low in the cortex and striatum of sham-operated mice (Fig. S1) but was significantly induced in ipsilateral cortex and striatum of the ischemic brain (Fig. 1f, h, Fig. S1). Besides, TRAF2 expression was mostly co-localized with the neuronal marker NeuN and the microglial marker Iba1 in the ischemic cortex and striatum at 24 h after reperfusion (Fig. 1f, g, h, i), and TRAF2^+^NeuN^+^ cells accounted for 56.0% and 71.2% of TRAF^+^ cells in the ipsilateral cortex and striatum (Fig. 1f, g), respectively. And TRAF2^+^Iba1^+^ cells accounted for 17.4% and 18.7% of TRAF^+^ cells in the ipsilateral cortex and striatum (Fig. 1h, i), respectively. These data suggest that neurons and microglia are two of the cellular sources for TRAF2 induction in the ischemic cortex and striatum.

### TRAF2 knockdown increased infarct volumes, cell death and neuroinflammation in the ischemic striatum

**Fig. 2:**
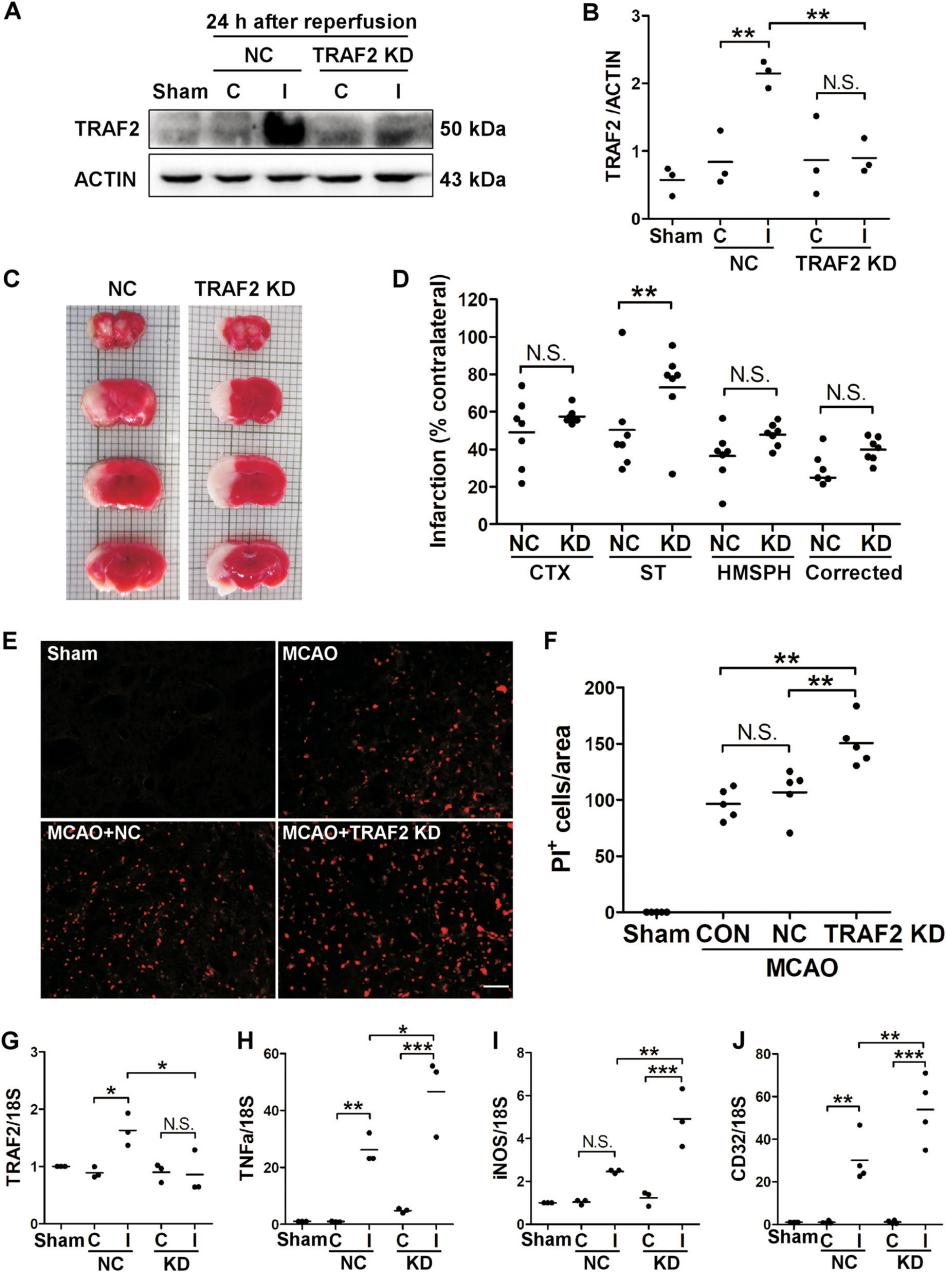
TRAF2 knockdown increased infarct volumes, brain cell death and neuroinflammation following MCAO. **a**–**b** Representative images of western blot analysis (**a**) and quantification (*n* = 3) (**b**) of TRAF2 expression in the contralateral and ipsilateral striatum of mice injected with NC shRNA or TRAF2 shRNA at 24 h after reperfusion. Lentiviruses expressing NC shRNA or TRAF2 shRNA were injected into the right striatum 14 days prior to MCAO. Then, MCAO was induced in the right hemisphere of lentivirus-injected mice. N.S.: not significant; **p < 0.01. Sham: sham-operated mice. NC: mice injected with lentivirus expressing NC shRNA. TRAF2 KD: mice injected with lentivirus expressing TRAF2 shRNA. C: contralateral side. I: ipsilateral side. **c** Representative TTC staining images of the brain slices from control or TRAF2 knockdown mice at 24 h after reperfusion. **d** TRAF2 knockdown significantly reduced acute infarction in the striatum at 24 h after MCAO (*n* = 7). N.S.: not significant; **p < 0.01. CTX: cortex. ST: striatum. HMSPH: hemisphere. Corrected: hemisphere infarction corrected for edema. NC: non-targeted control. KD: TRAF2 knockdown. **e** Representative images of PI staining in the ipsilateral striatum of sham-operated mice, mice without lentivirus infection (MCAO), mice ipsilaterally intrastriatally infected with NC shRNA lentivirus (MCAO + NC) or TRAF2 shRNA lentivirus (MCAO + TRAF2 KD) at 24 h after reperfusion. Scale bar: 50 μm. **f** Quantification of the amounts of PI^+^ cells in the ipsilateral striatum (*n* = 5). N.S.: not significant; **p < 0.01. CON: mice without lentivirus infection. **g–j** The mRNA levels of TRAF2 (g, *n* = 3), pro-inflammatory mediator TNFα (h, *n* = 3), iNOS (i, *n* = 3) and CD32 (j, *n* = 4) at 24 h after reperfusion in the striatum of mice injected with NC shRNA lentivirus (NC) or TRAF2 shRNA lentivirus (KD) in the right striatum. N.S.: not significant; *p < 0.05; **p < 0.01; ***p < 0.001. Sham: sham-operated mice

To investigate the role of TRAF2 following cerebral ischemia, lentiviruses expressing NC shRNA or an shRNA targeting murine TRAF2 were injected into the ipsilateral striatum 14 days prior to MCAO as we reported previously^19^. TRAF2 was increased in the ipsilateral striatum infected with NC shRNA lentivirus at 24 h after MCAO, and the induction was inhibited at the protein (Fig. 2a, b) and mRNA (Fig. 2g) levels in the ipsilateral striatum infected with TRAF2 shRNA lentivirus at 24 h after MCAO. The effects of striatal TRAF2 knockdown on the infarct volumes and cell death were examined. Compared with that of NC shRNA lentivirus-infected mice, the infarct volumes were increased in the ischemic cortex and striatum of TRAF2 shRNA lentivirus-infected mice (Fig. 2c, d) at 24 h after reperfusion. Moreover, TRAF2 knockdown remarkably increased the amount of PI^+^ cells in the ipsilateral striatum (Fig. 2e, f) at 24 h after reperfusion, indicating that knockdown of TRAF2 augmented MCAO-induced cell death.

Cerebral ischemia-induced cell death causes DAMPs releasing and triggers inflammatory events, such as microglia activation and the production of pro-inflammatory cytokines^25^, which in turn exacerbates cell death. Therefore, we further investigated whether TRAF2 knockdown promoted neuroinflammation by analyzing the expression of the pro-inflammatory markers. TNFα, iNOS and CD32 were increased in the ischemic striatum at 24 h after reperfusion in the mouse MCAO model (Fig. 2h–j), and striatal TRAF2 knockdown augmented ischemia-induced expression of these pro-inflammatory markers (Fig. 2h–j). These results suggest that TRAF2 induction following MCAO/reperfusion likely plays a protective role in the brain following cerebral ischemia by inhibiting ischemia-induced cell death and neuroinflammation.

### TRAF2 knockdown augmented microglial necroptosis in the in vitro ischemic condition

**Fig. 3:**
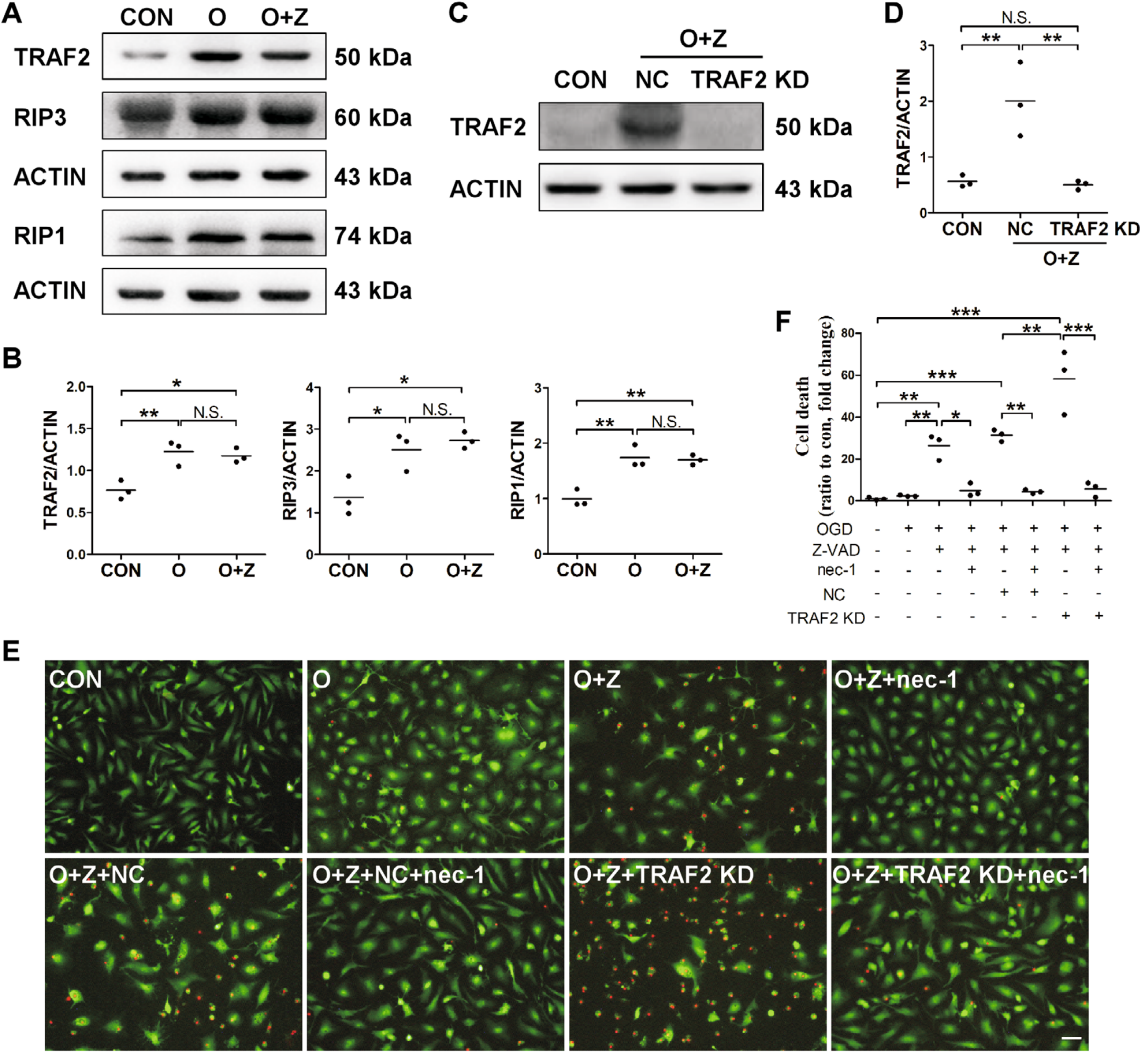
TRAF2 expression was increased in primary microglia treated with OGD neuron CM and Z-VAD, and TRAF2 knockdown augmented ischemic condition-induced microglial necroptosis. **a** Representative images of the western blot analysis of TRAF2, RIP3 and RIP1 in primary microglia at 12 h after exposure to normal neuron CM or OGD neuron CM in the absence or presence of the pan-caspase inhibitor Z-VAD. β-actin served as the internal reference. CON: control, normal neuron CM. O: OGD neuron CM. O + Z: OGD neuron CM plus Z-VAD. **b** Quantification of the western blot analysis of TRAF2, RIP3 and RIP1 (*n* = 3). N.S.: not significant; *p < 0.05; **p < 0.01. **c** Representative images of the western blot analysis of TRAF2 knockdown efficacy in primary microglia. **d** Quantification data showing that TRAF2 was knocked down (*n* = 3). N.S.: not significant; **p < 0.01. **e** Primary microglia were treated as indicated for 12 h and stained with PI and Calcein AM. Representative images captured by confocal microscopy were shown. Scale bar: 50 μm. **f** Quantification data of the amounts of PI^+^ cells were presented as ratio to control (*n* = 3). *p < 0.05; **p < 0.01; ***p < 0.001

Under ischemic stress, neuronal cells release DAMPs which activate receptors on microglia and prime microglia to the pro-inflammatory M1 phenotype, resulting in expression of pro-inflammatory mediators and inflammation-induced necrotic cell death in the presence of caspase inhibitor^2^. Microglia is one of the cellular sources for TRAF2 induction at 24 h after reperfusion in the mouse MCAO model (Fig. 1h, i). To investigate whether post-ischemic induction of TRAF2 impacts microglial survival, we utilized an in vitro microglial necroptosis model under mimicked ischemia condition. Primary microglia were treated with conditioned media (CM) from normal neurons (control CM) or from OGD-treated neurons (OGD neuron CM) for 12 h. We have shown that microglia treated with OGD neuron CM are primed to pro-inflammatory M1 phenotype^3^. OGD neuron CM *per se* did not induce cell death in primary microglia (Fig. 3e, f). In contrast, consistent with the previous report, microglial cell death was induced by OGD neuron CM in the presence of the pan-caspase inhibitor Z-VAD, as evident by the increase in the amounts of PI^+^ cells (Fig. 3e, f). Moreover, the protein levels of TRAF2, RIP1 and RIP3 were significantly increased when cells were incubated with OGD neuron CM (Fig. 3a, b). Treatment with the necroptosis inhibitor nec-1 suppressed the induction of microglial cell death (Fig. 3e, f). These results indicated that microglial necroptosis was induced by OGD neuron CM in the presence of Z-VAD. TRAF2 was knocked down in primary microglia with lentivirus expressing TRAF2 shRNA (Fig. 3c, d). TRAF2 knockdown remarkably exacerbated OGD neuron CM plus Z-VAD-induced cell death, and this effect was abrogated by nec-1 (Fig. 3e, f), suggesting that knocking down TRAF2 augmented ischemic condition-induced microglial necroptosis. TRAF2 knockdown did not induce cell death in primary microglia treated with OGD neuron CM in the absence of Z-VAD (Fig. S2a). Together, these results suggest that TRAF2 induction under the ischemic condition protected microglia against necroptosis.

### TRAF2 knockdown enhanced TNFα-induced necroptosis in hippocampal neuronal HT-22 cells

**Fig. 4:**
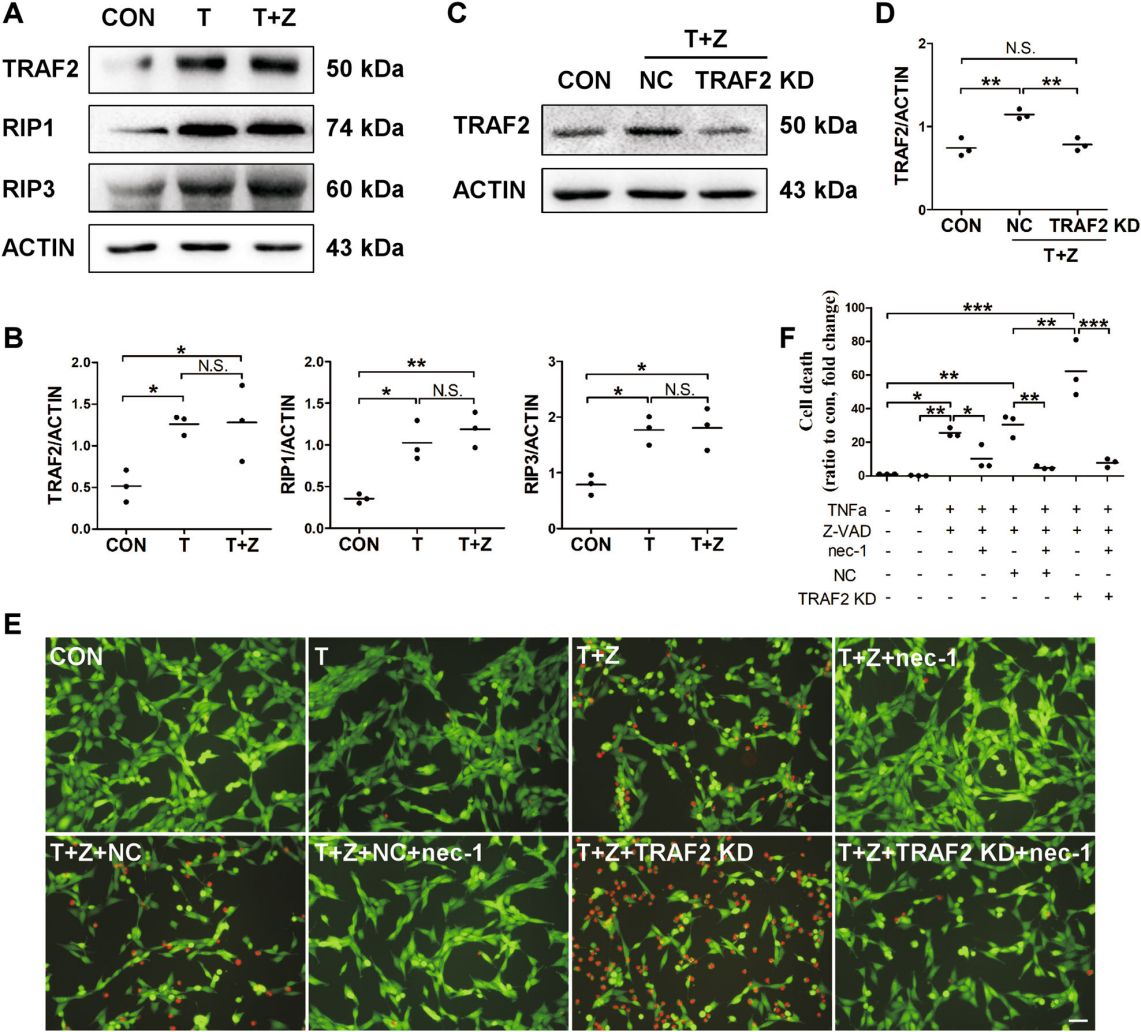
TRAF2 expression was increased in necroptotic HT-22 neurons treated with TNFα and Z-VAD, and TRAF2 knockdown augmented inflammation-induced neuronal necroptosis. **a** Representative images of the western blot analysis of TRAF2, RIP1 and RIP3 in hippocampal neuronal HT-22 cells at 12 h after treatment with vehicle or TNFα in the absence or presence of Z-VAD. β-actin served as the internal reference. CON: control. T: TNFα. T + Z: TNFα plus Z-VAD. **b** Quantification of the western blot analysis of TRAF2, RIP1 and RIP3 (*n* = 3). N.S.: not significant; *p < 0.05; **p < 0.01. **c** Representative images of the western blot analysis of TRAF2 knockdown efficacy in neuronal HT-22 cells. **d** Quantification data showing that TRAF2 was knocked down (*n* = 3). N.S.: not significant; **p < 0.01. **e** Neuronal HT-22 cells were treated as indicated for 12 h and stained with PI and Calcein AM. Representative images captured by confocal microscopy were shown. Scale bar: 50 μm. **f** Quantification data of the amounts of PI^+^ cells were presented as ratio to control (*n* = 3). *p < 0.05; **p < 0.01; ***p <0.001

Ischemia-induced over-activation of microglia leads to production of pro-inflammatory mediators, such as TNFα, and TNFα triggers cellular necroptosis when caspases are inhibited^2^. As we showed, neurons are one of the sources for TRAF2 induction following experimental stroke (Fig. 1f, g). To investigate whether the post-ischemic induction of TRAF2 functioned through a neuronal mechanism to impact brain pathology following cerebral ischemia, we utilized an in vitro neuronal necroptosis model. Hippocampal neuronal HT-22 cells were treated with vehicle or TNFα for 12 h. TNFα treatment alone did not induce neuronal cell death, unless apoptosis was suppressed by Z-VAD (Fig. 4e, f). Compared with that in cells treated with vehicle, the protein levels of TRAF2, RIP1 and RIP3 were significantly increased in cells treated with TNFα (Fig. 4a, b). Moreover, the addition of nec-1 inhibited the induction of cell death in TNFα plus Z-VAD-treated HT-22 cells (Fig. 4e, f). These results indicated that neuronal necroptosis was induced in HT-22 cells treated with TNFα and Z-VAD. TRAF2 was knocked down in HT-22 cells, and the knockdown efficacy was confirmed by western blot (Fig. 4c, d). TRAF2 knockdown markedly augmented TNFα plus Z-VAD-induced neuronal cell death, and this effect was abolished by nec-1 (Fig. 4e, f), indicating that knocking down TRAF2 exacerbated inflammation-induced neuronal necroptosis. TRAF2 knockdown did not induce cell death in HT-22 neurons treated with TNFα in the absence of Z-VAD (Fig. S2b). We also examined the effect of TRAF2 knockdown on cell death in primary neurons subjected to OGD treatment. OGD-induced neuronal death either in the absence or presence of Z-VAD was not inhibited by nec-1 (Fig. S3), suggesting that necroptosis does not account for neuronal death induced by OGD, regardless of the presence or absence of Z-VAD. Moreover, TRAF2 knockdown did not affect OGD-induced neuronal death in the presence or absence of Z-VAD (Fig. S3). Together, our results suggest that neuronal induction of TRAF2 under the ischemia-induced inflammatory condition may protect neurons against necroptosis.

### RIP1, RIP3 and MLKL levels were increased in vivo and the complex formation between TRAF2 and MLKL was increased in the ischemic striatum following MCAO

**Fig. 5:**
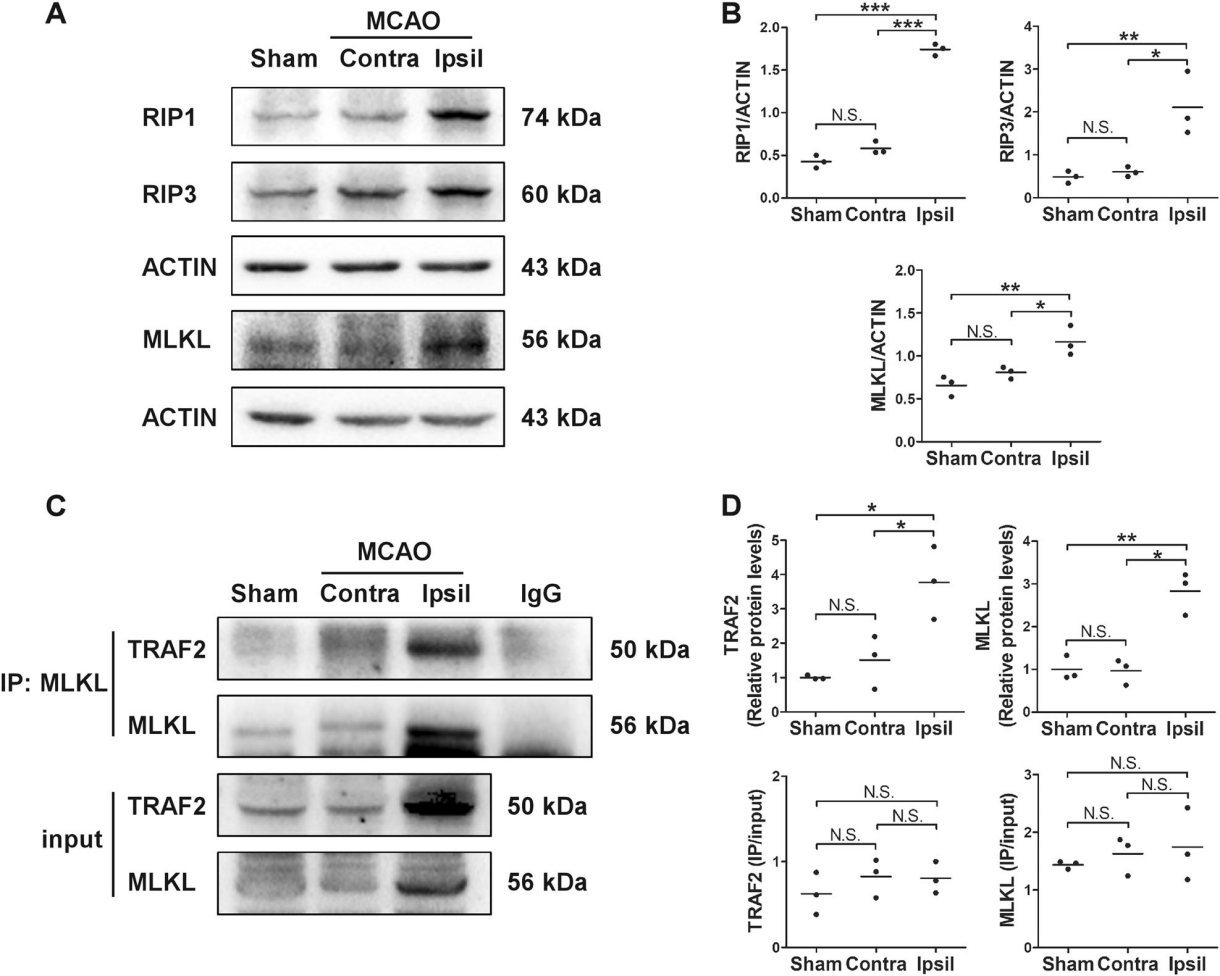
Expression of RIP1, RIP3 and MLKL were increased and the complex formation between TRAF2 and MLKL was increased in the ischemic striatum following MCAO. **a** Representative images of the western blot analysis of RIP1, RIP3 and MLKL in the striatum of sham-operated or MCAO mice at 24 h after reperfusion. Contra: contralateral side. Ipsil: ipsilateral side. **b** Quantification data showing that RIP1, RIP3 and MLKL were significantly increased in the ischemic striatum at 24 h after MCAO (*n* = 3). N.S.: not significant; *p < 0.05; **p < 0.01; ***p < 0.001. **c** Total proteins in the lysates of striatum were incubated with anti-MLKL antibody and protein A/G agarose beads, and TRAF2 and MLKL levels in the lysates (input) and in the immunoprecipitated proteins (IP) were analyzed by western blot. Representative images were shown. **d** Quantification data of the protein levels of immunoprecipitated TRAF2 and MLKL, showing that the complex formation between TRAF2 and MLKL was increased in the ischemic striatum. The IP quantification data were also normalized to the total protein levels of TRAF2 and MLKL, respectively (*n* = 3). N.S.: not significant; *p < 0.05; **p < 0.01

Using the in vitro cellular models, we showed that the levels of RIP1 and RIP3, two critical proteins involved in the induction of necroptosis, were increased in primary microglia and neuronal cells responding to ischemic or inflammatory insults. Next, we assessed the protein levels of RIP1, RIP3 and another necroptosis-related protein MLKL in the striatum in mouse MCAO model at 24 h after reperfusion. Consistent with the in vitro results, the in vivo levels of RIP1 and RIP3 were significantly increased in the ipsilateral striatum, compared with that in the contralateral striatum of mice received MCAO or in the striatum of sham-operated mice (Fig. 5a, b). Moreover, MLKL level was also increased in the ischemic striatum (Fig. 5a, b). These results suggest that necroptosis is likely induced in the ischemic brain following cerebral ischemia. Recently, TRAF2 has been reported to be an important suppressor for necroptosis^12,13^ through interacting with MLKL^13^. Since the expression of TRAF2 was induced following cerebral ischemia (Fig. 1), and TRAF2 knockdown augmented ischemic and inflammatory condition-induced cell death both in vivo (Fig. 2e, f) and in vitro (Figs. 3e, f and 4e, f), we further investigated whether TRAF2 played a protective role in cell death following experimental ischemic stroke by interacting with MLKL. To show this we performed co-immunoprecipitation. The complex formation between TRAF2 and MLKL was apparently increased in the ipsilateral striatum at 24 h after reperfusion, compared with that in the contralateral striatum and in the striatum of sham-operated mice (Fig. 5c, d). Considering that the total protein levels of TRAF2 and MLKL were also increased, the IP quantification was further normalized to the total protein level (Fig. 5d). The results suggest that the increased complex formation between TRAF2 and MLKL in the ischemic striatum was not due to higher affinity between the proteins, but likely attributed to increased expression of TRAF2. To conclude, TRAF2 likely serves as a suppressor for cerebral ischemia-induced necroptosis through interaction with MLKL.

### Inhibiting necroptosis abrogated the exacerbating effect of TRAF2 knockdown on cerebral ischemia-induced cell death

**Fig. 6:**
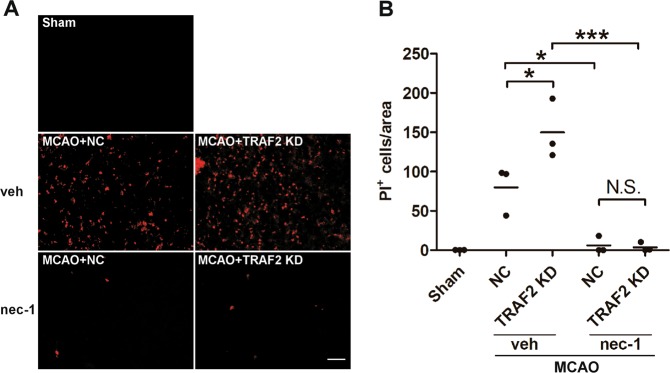
Administration of nec-1 abrogated the exacerbating effect of TRAF2 knockdown on ischemia-induced cell death following MCAO. **a** Representative images of PI staining in the ipsilateral striatum at 24 h after reperfusion. Scale bar: 50 μm. Sham: sham-operated mice. NC: mice injected with lentivirus expressing NC shRNA. TRAF2 KD: mice injected with lentivirus expressing TRAF2 shRNA. Veh: Vehicle. **b** Quantification of the amounts of PI^+^ cells in the ipsilateral striatum (*n* = 3). N.S.: not significant; *p < 0.05; ***p < 0.001

Nec-1 is a specific inhibitor of necroptosis, and it has been shown to protect against MCAO-induced brain injury^4^. To further show that TRAF2 knockdown exacerbates ischemic damage via enhanced necroptosis, we investigated whether nec-1 could rescue the cell death augmented by TRAF2 knockdown following cerebral ischemia. NC shRNA or TRAF2 shRNA lentiviruses were injected into the ipsilateral striatum 2 weeks prior to MCAO, and vehicle or nec-1 was administrated intracerebroventricularly just before MCAO. Compared with that in the ipsilateral striatum of NC shRNA lentivirus-injected mice, the amount of PI^+^ cells were significantly increased in the ischemic striatum of TRAF2 shRNA lentivirus-injected mice (Fig. 6a, b), whereas administration of nec-1 remarkably abolished cerebral ischemia-induced cell death, both in control and TRAF2 knockdown mice. These results suggest that TRAF2 protects against cerebral ischemic damage by suppressing necroptotic cell death following experimental stroke.

## Discussion

In the present study, we showed that the expression of TRAF2 was significantly induced in the ischemic brain at 24 h following MCAO. In addition, the post-ischemic induction of TRAF2 protected microglia and neurons against necroptotic cell death, possibly through enhanced interaction with MLKL. Our results suggest that TRAF2 is a novel regulator of cerebral ischemic injury.

TRAF family members are adaptor proteins for the tumor necrosis factor receptor family, and are important regulators of cell survival, cell death and cellular response to pathological stresses^26^. In the context of ischemia/reperfusion (I/R) stress, TRAF1 expression is induced responding to hepatic I/R injury, and its deficiency is protective whereas its overexpression aggravates I/R-induced liver injury^27^. Additionally, TRAF1 expression is induced at 6 h after ischemic stroke, and up-regulation of neuronal TRAF1 augments neuronal death and exacerbates ischemic lesions, whereas its deficiency is neuroprotective^14^. Neuronal expression of TRAF3 is induced in response to ischemic stroke, and overexpression of TRAF3 in neurons exacerbates neuronal loss and increases infarct volumes, whereas TRAF3 knockout protects mice from cerebral ischemia^28^. These results suggest a harmful role of TRAF family members in I/R injury. In addition, using an in vitro OGD/reperfusion model, Su et al.^29^ has reported that microglial TRAF2 knockdown inhibited microglia-mediated secretion of NFκB and IL17 and reduced I/R-induced neuronal apoptosis, suggesting a harmful role of TRAF2 in cerebral ischemia. However, TRAF2 is reported to play a protective role in cardiac ischemia, as TRAF2 expression in the heart is induced following pressure overload and attenuates myocardial infarction^30^. Cardiac-restricted mild overexpression of TRAF2 confers cytoprotective effects and protects the heart against cardiac ischemic injury^31,32^. Whether TRAF2 plays a protective/detrimental role in cerebral ischemia in vivo has not been investigated. We showed here that TRAF2 was induced in the brain following cerebral ischemia. To understand its role in ischemic brain injury, TRAF2 was knocked down using lentiviral vector-mediated delivery of shRNA. TRAF2 knockdown enlarged infarct volume and increased cell death. In addition, the expression of pro-inflammatory mediators TNFα, iNOS and CD32 were further increased in the ischemic striatum when TRAF2 was knocked down. These results indicate a beneficial role of TRAF2 induction in the brain upon ischemic stress. Unexpectedly, intrastriatal injection of TRAF2 shRNA-expressing lentivirus also increased infarct volumes in the cortex and hemisphere, compared with injection of lentivirus expressing NC shRNA, but viral leakage into the cortex was not observed. These unexpected results were consistent with the precious reports and may be partially attributed to standby effects^19^, such as the indirect effect on the survival of noninfected neurons by lentivirus-infected neurons^33^. The mechanism underlying TRAF2 induction following cerebral ischemia needs further investigation.

It has been reported that TRAF2 plays a protective role in ischemia and inflammatory mediator-induced injury through suppressing apoptotic and necroptotic cell death^12,30,34^. Both apoptosis and necroptosis are involved in ischemic brain damage, depending on the duration time and severity of the injury^10^, while the inhibitor of necroptosis provides neuroprotection in an extended time window^4^. Suppressing necroptosis significantly reduces inflammatory response and infarct volumes, and improves stroke outcomes^4,5,8,9^. We showed that brain ischemia-induced TRAF2 was localized predominantly in neurons and microglia in the ipsilateral cortex and striatum. Thus, we investigated the role of TRAF2 in microglial and neuronal cell death using the in vitro cellular models. Microglial activation accompanies neuronal cell death following stroke, as microglia respond to DAMPs such as HMGB1 released from ischemic neurons^25^. In turn, activated microglia release neurotoxic factors which augment the death of injured neurons^25^. Moreover, microglial necroptosis has been reported to contribute to neuroinflammation by releasing various pro-inflammatory mediators^35^, likely further exacerbating ischemic damage. To investigate the effects of TRAF2 on microglial death under the ischemic condition, mouse primary microglia were treated with OGD neuron CM or normal neuron CM. The expression of TRAF2 in microglia was induced upon OGD neuron CM treatment. Microglial cell death was not observed, unless the addition of the pan-caspase inhibitor Z-VAD, whereas the presence of the necroptosis inhibitor nec-1 abrogated the cell death, indicating that necroptosis was induced in OGD neuron CM-treated microglia when apoptosis was inhibited. TRAF2 knockdown in primary microglia further enhanced microglial necroptosis induced by OGD neuron CM in the presence of Z-VAD, which was also abolished by nec-1. The results indicate that TRAF2 induction under the in vitro ischemic condition protects against microglial necroptosis. Necroptotic microglia has been reported to release various pro-inflammatory mediators and lead to neuroinflammation^35^. Consistently, in the in vivo mouse MCAO model, we observed that TRAF2 knockdown enhanced cerebral ischemia-induced neuroinflammation.

To investigate the role of TRAF2 in neuronal necroptosis under inflammatory condition, mouse hippocampal neuronal HT-22 cells were treated with TNFα and Z-VAD. Similarly, TRAF2 was induced in HT-22 cells upon TNFα treatment. Neuronal cell death was not observed unless the addition of Z-VAD, whereas nec-1 remarkably abrogated the cell death induced by TNFα plus Z-VAD. These data indicate that necroptosis is induced in TNFα-treated HT-22 when caspases are inhibited. TRAF2 knockdown in HT-22 cells further increased TNFα plus Z-VAD-induced neuronal cell death, and this was abolished by nec-1. Taken together, these in vitro results indicate that TRAF2 induction responding to ischemic and inflammatory insults has beneficial effects in protecting against microglial and neuronal necroptosis. These results are in line with our in vivo results that striatal TRAF2 knockdown significantly increased cell death in the ischemic striatum following MCAO, whereas pre-administration of nec-1 remarkably blocked the induction of cell death. Since nec-1 is a specific necroptosis inhibitor, these results demonstrate that TRAF2 plays a protective role by inhibiting necroptotic cell death following experimental stroke. Inconsistent with our in vitro and in vivo findings, it has been reported that microglial TRAF2 knockdown decreased OGD-induced neuronal apoptosis in vitro^29^. This discrepancy might be caused by the different cellular models utilized, as in our model we studied the effect of neuronal TRAF2 knockdown on inflammation-induced neuronal HT-22 cell death. Moreover, we examined the effect of TRAF2 knockdown on neuronal death induced by OGD in primary neurons. First, we observed that nec-1 did not inhibit OGD-induced neuronal death, regardless of the presence or absence of Z-VAD. These results suggest that OGD alone or OGD plus Z-VAD does not induce neuronal necroptosis. Consistently, it has been reported that nec-1 across a wide range of concentrations did not protect primary neurons^36^ or the neuronal cell line^37^ against OGD insults. Collectively, these results suggest that OGD induces neuronal death via other mechanisms rather than necroptosis. Moreover, we showed that TRAF2 knockdown did not augment cell death induced by OGD in primary neurons with/without Z-VAD, suggesting that TRAF2 may only regulate necroptotic cell death.

The induction of necroptosis requires RIP1, RIP3 and MLKL^6,38^. It has been reported that RIP1 and RIP3 are increased in CA1 hippocampus in a rat model of transient global cerebral ischemia^6^ and in the developing cortex and hippocampus in a neonatal rat model of hypoxia/ischemia^39^, but MLKL protein level is unaltered in the neonatal hypoxia/ischemia model^39^. To investigate the molecular mechanisms underlying the protective role of TRAF2 in cerebral ischemia, we assessed the levels of RIP1, RIP3 and MLKL following MCAO. The protein levels of RIP1, RIP3 and MLKL were significantly increased in the ischemic striatum at 24 h after reperfusion. In accordance with the in vivo results, microglial and neuronal RIP1 and RIP3 levels were also raised reacting to ischemic and inflammatory insults in vitro. It has been reported that TRAF2 constitutively associates with MLKL, whereas TNFα plus Z-VAD plus cycloheximide treatment induces their dissociation in MEFs and L929 cells^13^, which increases the association of MLKL with RIP3 and induces necroptosis. However, Guo et al. has reported that TRAF2 and MLKL constitutively interacts with each other in cardiomyocytes that treated with TNFα plus Z-VAD^30^. In consistent, we also did not observe the disruption of TRAF2-MLKL binding in the ipsilateral striatum following cerebral ischemia. Co-immunoprecipitation data showed that post-MCAO induction of TRAF2 resulted in enhanced complex formation between TRAF2 and MLKL in the ipsilateral striatum. Thus, TRAF2 induction likely inhibits cerebral ischemia-induced necroptosis through increased complex formation with MLKL, consequently inhibiting the association between MLKL and RIP3. Further investigation is needed to confirm the decreased interaction between RIP3 and MLKL following experimental stroke.

In conclusion, our study showed that TRAF2 was induced in the brain following experimental stroke. Suppressing TRAF2 induction amplified the infarct volume and increased cell death likely via the necroptosis mechanisms. Our results also showed that TRAF2 formed more complex with MLKL following experimental stroke, suggesting that TRAF2 may inhibit necroptosis by abating the association between MLKL and RIP3. Taken together, our results indicate TRAF2 as a novel regulator of cerebral ischemic injury.

## Supplementary information

Figure S1

Figure S2

Figure S3

Supplementary figure legends
