# Supplementary figures and images for: TRAF2 protects against cerebral ischemia-induced brain injury by suppressing necroptosis
Source: Cell Death Dis. 2019 Apr 15;10(5):328. doi: 10.1038/s41419-019-1558-5 (PMC6465397; doi:10.1038/s41419-019-1558-5)

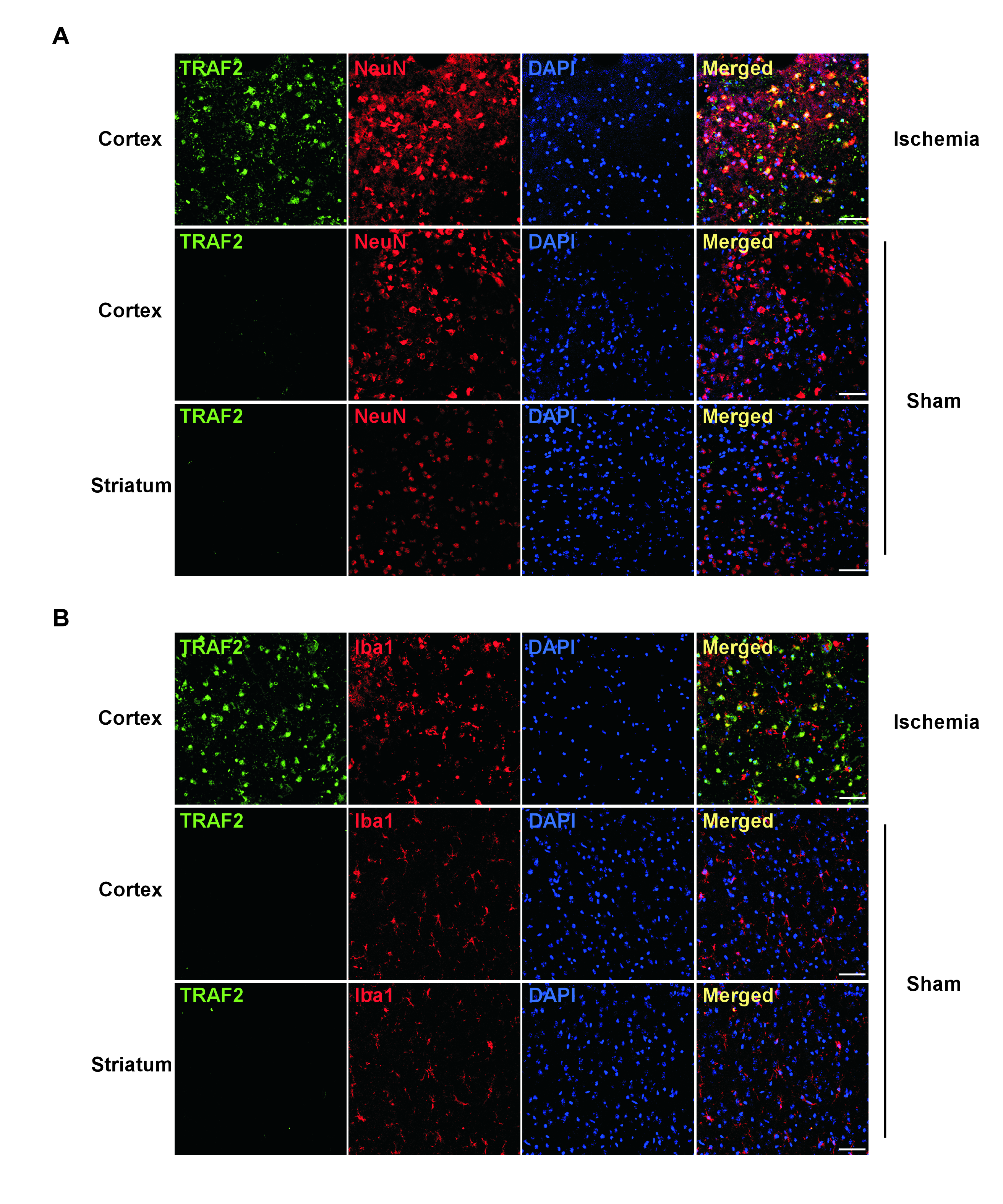

Supplement: Supplementary file 1 — Figure S1 [file 41419_2019_1558_MOESM1_ESM.tif]

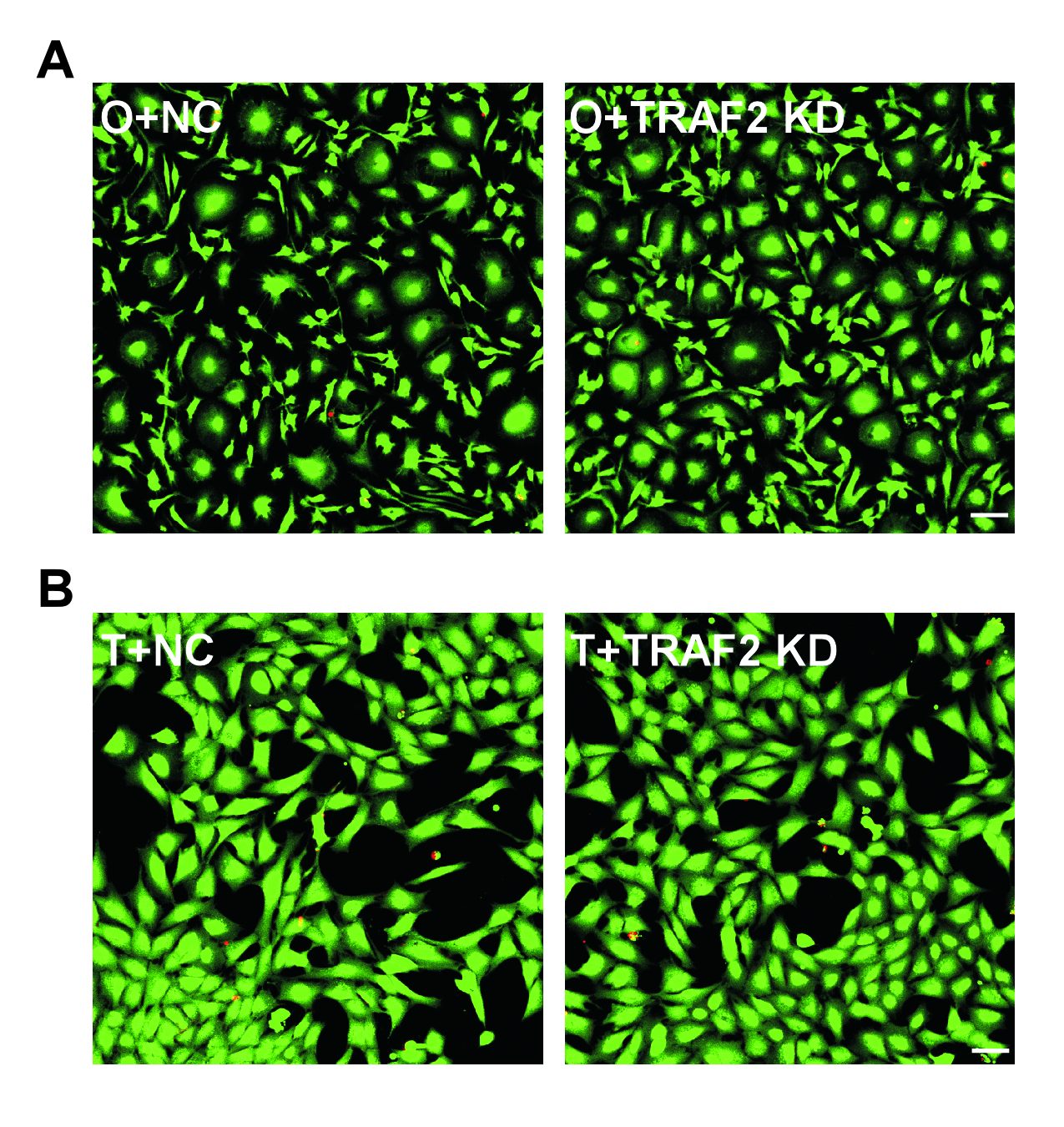

Supplement: Supplementary file 2 — Figure S2 [file 41419_2019_1558_MOESM2_ESM.tif]

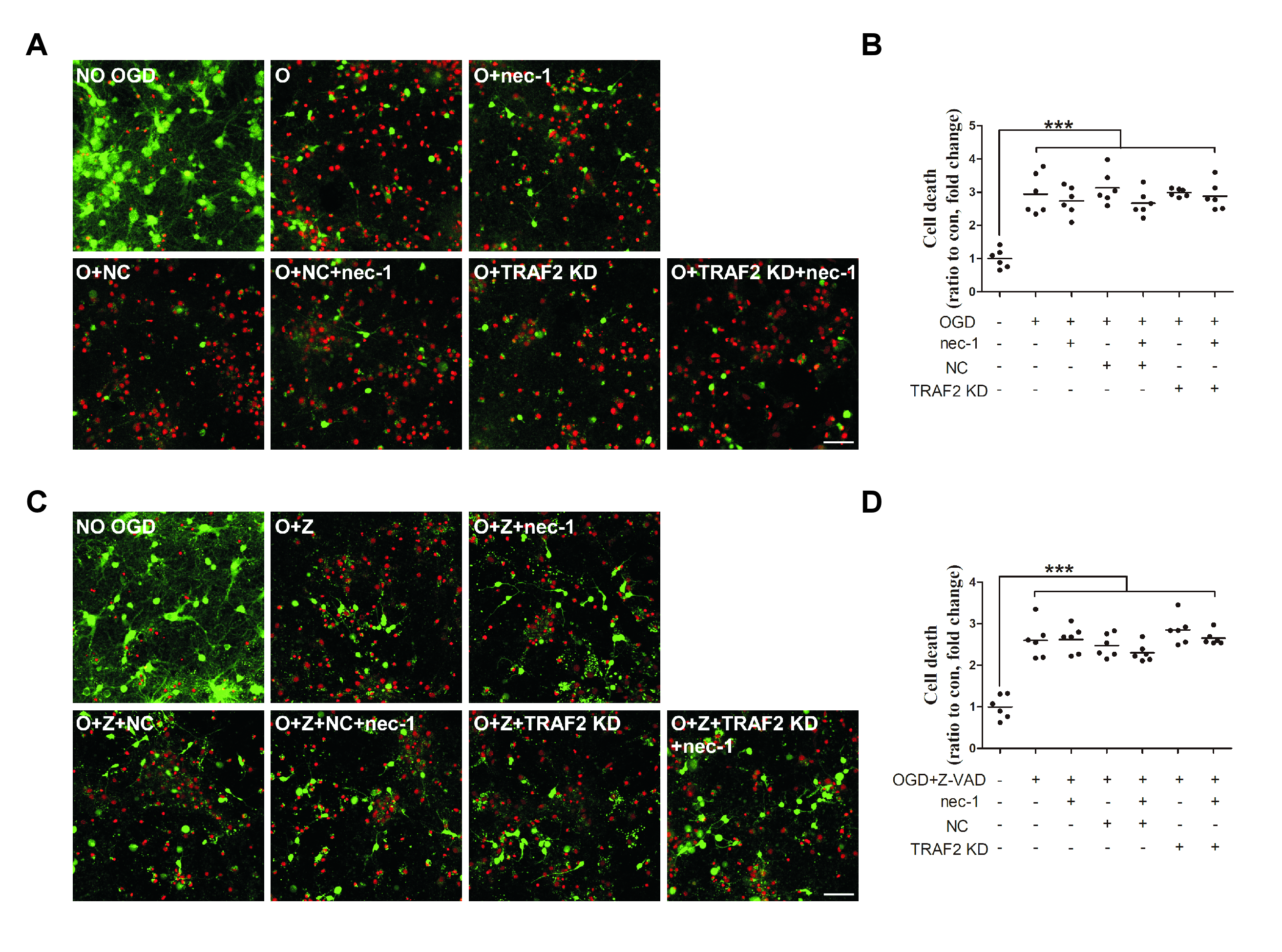

Supplement: Supplementary file 3 — Figure S3 [file 41419_2019_1558_MOESM3_ESM.tif]
