# Supplementary material for: TRAF2 protects against cerebral ischemia-induced brain injury by suppressing necroptosis
Source: Cell Death Dis. 2019 Apr 15;10(5):328. doi: 10.1038/s41419-019-1558-5 (PMC6465397; doi:10.1038/s41419-019-1558-5)
Supplement: Supplementary file 4 — Supplementary figure legends [file 41419_2019_1558_MOESM4_ESM.docx]

**Supplementary figure legends:**

**Fig. S1. Expression patterns of TRAF2 in neurons and microglia in the cortex and striatum of sham-operated mice.** (A) Co-localization of TRAF2 with the neuronal marker NeuN in the ipsilateral cortex and striatum at 24 h after sham operation. Scale bar: 50 μm. (B) Co-localization of TRAF2 with the microglial marker Iba1 in the ipsilateral cortex and striatum at 24 h after sham operation. Scale bar: 50 μm. The ischemic cortex was used as a positive control since TRAF2 was induced at 24 h after MCAO/reperfusion.

**Fig. S2. TRAF2 knockdown did not induce cell death in primary microglia treated with OGD neuron CM alone and in HT22 neurons treated with TNFα in the absence of Z-VAD.** (A) PI and Calcein AM staining in primary microglia infected with lentivirus expressing NC shRNA or TRAF2 shRNA for 4-5 days and then treated with OGD neuron CM. Notably, some microglia shifted from the bipolar morphology to the activated amoeboid morphology following OGD neuron CM treatment. Scale bar: 50 μm. O: OGD neuron CM. (B) PI and Calcein AM staining in neuronal HT-22 cells infected with lentivirus expressing NC shRNA or TRAF2 shRNA and then treated with TNFα. Scale bar: 50 μm. T: TNFα.

**Fig. S3.** **TRAF2 knockdown did not affect OGD-induced cell death in primary neurons either in the absence or presence of Z-VAD.** (A) PI and Calcein AM staining in primary cortical neurons infected with lentivirus expressing NC shRNA or TRAF2 shRNA and then subjected to OGD for 2 h with/without nec-1. Scale bar: 50 μm. (B) Quantification data of the numbers of PI^+^ cells were presented as ratios to control (n = 6). ***: *p*<0.001. (C) PI and Calcein AM staining in primary cortical neurons infected with lentivirus expressing NC shRNA or TRAF2 shRNA and then subjected to OGD in the presence of Z-VAD with/without nec-1. Scale bar: 50 μm. (D) Quantification data of the numbers of PI^+^ cells were presented as ratios to control (n = 6). ***: *p*<0.001.
